# A vascular component to the selective vulnerability of the medial temporal lobe to tau pathology

**DOI:** 10.1093/braincomms/fcag301

**Published:** 2026-08-31

**Authors:** Vincent Doyon, Félix Janelle, Monica Sean, Davy Vanderweyen, Samantha Côté, Julia Huck, Pascal Tétreault, Jean-François Lepage, Jose Gutierrez, Christian Bocti, Tamàs Fülöp, Alexa Pichet-Binette, Kevin Whittingstall

**Affiliations:** Department of Medical Imaging and Radiation Sciences, Université de Sherbrooke, Sherbrooke J1H 5N4, Canada; Department of Medical Imaging and Radiation Sciences, Université de Sherbrooke, Sherbrooke J1H 5N4, Canada; Department of Surgery, Université de Montréal, Montréal H3T 1J4, Canada; Department of Medical Imaging and Radiation Sciences, Université de Sherbrooke, Sherbrooke J1H 5N4, Canada; Department of Medical Imaging and Radiation Sciences, Université de Sherbrooke, Sherbrooke J1H 5N4, Canada; Department of Psychology, Bishop’s University, Sherbrooke J1M 1Z7, Canada; Department of Radiology, University of Calgary, Calgary T2N 1N4, Canada; Department of Medical Imaging and Radiation Sciences, Université de Sherbrooke, Sherbrooke J1H 5N4, Canada; Department of Anesthesiology, Université de Sherbrooke, Sherbrooke J1H 5N4, Canada; Department of Pediatrics, Université de Sherbrooke, Sherbrooke J1H 5N4, Canada; Centre de Recherche du Centre Hospitalier Universitaire de Sherbrooke, Sherbrooke J1H 5N4, Canada; Department of Neurology, University of Miami Miller School of Medicine, Miami 33136, USA; Department of Medicine, Université de Sherbrooke, Sherbrooke J1H 5N4, Canada; Department of Medicine, Université de Sherbrooke, Sherbrooke J1H 5N4, Canada; Department of Pharmacology and Physiology, Université de Montréal, Montréal H3T 1J4, Canada; Centre de Recherche de L’Institut Universitaire de Gériatrie de Montréal, Montréal H3W 1W6, Canada; Department of Medical Imaging and Radiation Sciences, Université de Sherbrooke, Sherbrooke J1H 5N4, Canada; Centre de Recherche du Centre Hospitalier Universitaire de Sherbrooke, Sherbrooke J1H 5N4, Canada; Department of Diagnostic Radiology, Université de Sherbrooke, Sherbrooke J1H 5N4, Canada

**Keywords:** brain artery, vascular, circle of Willis, aging, Alzheimer’s disease

## Abstract

The medial temporal lobe is widely recognized as an early site of pathological tau deposition, a central feature in the development of cognitive symptoms. As of now, the biological mechanisms underlying this selective vulnerability of the medial temporal lobe are not yet understood. Here, we tested whether alterations in medial temporal lobe arterial structure are associated with early tau accumulation and cognitive decline. We first applied a novel imaging methodology (super-selective arterial spin labelling) to map vascular supply to the medial temporal lobe. Next, we cross-sectionally investigated the relationship between medial temporal lobe artery diameters and tau burden, derived from magnetic resonance angiography and positron emission tomography imaging, in 151 cognitively unimpaired individuals (mean age: 67.6 years old). Finally, we examined whether artery diameter was correlated with the rate of cognitive decline in a separate cohort of 43 individuals (mean age: 74.2 years old). We found that the blood supply to the medial temporal lobe depended more on arteries from the anterior circulation than the posterior circulation. Individuals with small anterior choroidal artery diameters tended to show higher medial temporal lobe tau burden (r = −0.26, *P* = 0.001) and faster rates of cognitive decline (r = −0.40, *P* = 0.012). These results remained significant after adjusting for potential confounding factors such as amyloid burden. Our results show that anterior choroidal artery diameter may be an amyloid-independent determinant of tau pathology and cognitive decline. This suggests that the selective vulnerability of the medial temporal lobe may emerge from subpar perfusion from the anterior circulation.

## Teaser

Doyon *et al*. found that individuals with narrower anterior choroidal arteries, which supply the anterior portion of the medial temporal lobe, have higher tau burden and experience faster cognitive decline.

## Graphical Abstract

**Graphical Abstract:**
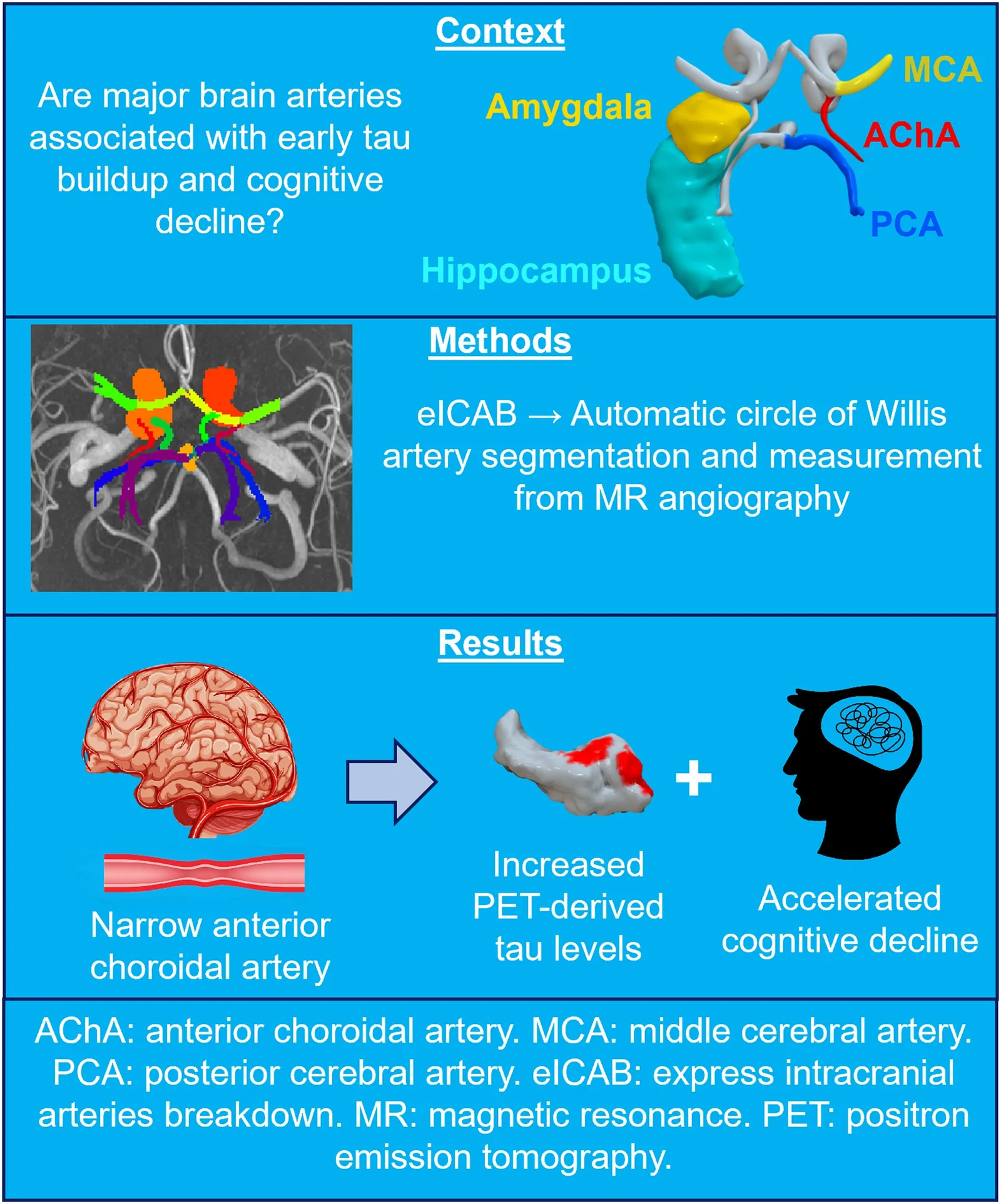

## Introduction

The medial temporal lobe (MTL) is a critical brain region encompassing structures that are essential for memory formation, long-term storage and spatial navigation.^1,2^ During normal aging, subregions of the MTL such as the hippocampus, the amygdala and the entorhinal cortex are among the first to undergo detrimental changes,^3^ most notably the accumulation of pathological tau proteins.^4^ In a physiological context, tau is involved in axonal transport and structural integrity; however, under pathological conditions, it becomes hyperphosphorylated and tends to aggregate and form neurofibrillary tangles, which are toxic to neurons.^5^ It has been shown that tau levels in the MTL of cognitively unimpaired (CU) individuals increase the risk of future cognitive decline,^6-8^ highlighting the importance of identifying the factors driving tau accumulation. Individuals with elevated amyloid beta (Aβ) tend to accumulate tau more rapidly than those without, suggesting that Aβ may act as a key driver of tau pathology and tau-mediated neurodegeneration.^9,10^ However, because Aβ and tau pathologies initially accumulate in different brain regions^11^ and tau aggregation can precede Aβ deposition,^12,13^ the idea that tau pathology is solely driven by Aβ pathology is controversial.^14^ Regional gene expression associated with tau pathology is thought to be the main cause behind the MTL’s ‘selective vulnerability’, but other mechanisms may also play a role.^15^

Maintaining adequate cerebral blood flow is essential for overall brain health, cognitive function and prevention of neurological disorders. In humans, oxygen-rich blood reaches the brain via the Circle of Willis, a ring-like arterial structure located at the base of the brain. Human autopsy and animal studies have shown that luminal narrowing of the Circle of Willis arteries is associated with increased tau burden,^16-19^ presumably due to chronic hypoperfusion.^20-23^ Moreover, *in vivo* human imaging studies show that the vascular architecture supplying blood to the MTL is an important determinant of cognitive health,^24,25^ thus raising the possibility that arterial structure supplying the MTL may be an important component of its selective vulnerability. The so-called ‘vascular’ theory of selective vulnerability was first proposed over a century ago,^26^ yet has since then been largely ignored and experimental data linking it to tau pathology and cognition remain limited.

To investigate this, we used a fully automated method to analyse the Circle of Willis arteries on magnetic resonance angiography (MRA) images. With this, we correlated their lumen diameters with (i) tau burden in the MTL, measured with PET in cognitively unimpaired individuals (CU, *N* = 151); and (ii) longitudinal measures of the rate of cognitive decline in a separate cohort of individuals with mild cognitive impairment (MCI, *N* = 43). As a complementary experiment, we sought to establish the perfusion territories of MTL arteries using super-selective arterial spin labelling (ss-ASL).

## Materials and methods

### Artery-specific supply of the MTL

We performed ss-ASL in five young participants (mean age = 27.2 ± 4.5 years old, two males and three females). Imaging protocol was approved by the review board of the Centre Hospitalier Universitaire de Sherbrooke, and all participants gave their written informed consent for this study. Each participant underwent a 40 min scan on a 3T Ingenia Phillips scanner equipped with a 32-channel head coil (Philips Healthcare, Best, Netherlands). The imaging session included a time-of-flight sequence centered on the pons (field of view (FOV) = 320 × 320 × 162, TR = 23 ms, TE = 3.454 ms, voxel size of 0.625 × 0.625 × 0.750 mm, total scan time = 3 minutes 52 seconds), two pseudo-continuous ss-ASL sequences for which the FOV was centered on the MTL and a high-resolution T1-weighted image of the whole brain (FOV = 224 × 224 × 160, TR = 7.9 ms, TE = 3.5 ms, voxel size of 1 × 1 × 1 mm, total scan time = 4 minutes 19 seconds).

For the ss-ASL sequences (FOV = 80 × 80 × 10, TR = 5100 ms, TE = 16.077 ms, voxel size of 3 × 3 × 5 mm, post-label delay = 1700ms, label duration = 1650 ms, number of pairs of control/label images: 60, scan time for a single tagging = 10 min 22 s), blood from the anterior circulation was labelled about 60 mm down the Circle of Willis in the cervical segment of the internal carotid artery (ICA). For the posterior circulation, labelling was performed ∼75 mm down the Circle of Willis in the V2 segment of the vertebral arteries (VA). To tag both vertebral arteries and thus increase the tagging efficiency, labelling selectivity was removed along the left-right axis by omitting a magnetic gradient as previously described in Murazaki *et al*. (2022).^27^ For one participant, two ss-ASL images of the vertebral arteries were obtained and summed during preprocessing due to a low signal-to-noise ratio in a single acquisition.

To ensure that tagged blood from the anterior circulation did not travel to the posterior cerebral artery (PCA) through the posterior communicating artery and thus contaminate the signal, only participants with hypoplastic posterior communicating arteries on their MRA images were included.

All MRI images were converted from DICOM to NIFTI format using dcm2niix (version v1.0.20220505). A combination of AFNI (version 24.3.00) and FSL tools (version 6.0.7.12) was used for the following steps. The ss-ASL images were first registered to the T1-weighted image. Brain segmentation was performed on the high-resolution T1-weighted images using FastSurfer.^28^ The hippocampus was delineated into three subregions (head, body and tail) using k-means clustering. While this method does not account for the anatomical boundaries of each hippocampal subregion, it effectively separates the hippocampus into three regions along its anterior–posterior axis.

To standardize ASL units, each voxel intensity was divided by the mean intensity value of a composite region typically perfused by the tagged artery. The composite regions included the grey matter of the superior and medial temporal cortex, supramarginal cortex, insula and putamen for the ICA. The composite region for the posterior circulation consisted of the grey matter of the precuneus and lingual cortex. Resulting ASL image units therefore represented the percentage of perfusion of the composite region. Clustering was then performed on ASL images to reduce noise level with the following parameters: threshold value = 0.4, minimal number of voxels to be considered a cluster = 20, face-wise connectivity.

To determine the supply dependency of each MTL region, a voxel with a value of 40% or over was assumed to be irrigated by the tagged artery. The 40% threshold represents the upper limit at which nearly all grey matter voxels were perfused by either the internal carotid artery or the basilar artery. The proportion of irrigation within each MTL region was calculated as the number of irrigated voxels divided by the total number of voxels in that region.

### Relationship between tau and artery size

The correlation between tau levels and artery diameter was analysed using the OASIS dataset,^29^ which includes MRI and PET images from CU participants. MRI acquisitions were conducted using a Biograph mMR or a TrioTim scanner equipped with a 16-channel head coil. For PET scans, a Biograph mMR, Biograph 40 PET/CT or an ECAT HRplus 962 scanner was used. To measure tau levels, 370 MBq of Flortaucipir was used as a tracer and images were acquired 75 min post-injection for a duration of 30 min. Amyloid burden was assessed with AV45 (370 MBq) or PIB tracer (222 MBq to 740 MBq). Other relevant information regarding PET and MRI acquisition procedures is detailed at https://sites.wustl.edu/oasisbrains/home/oasis-3/.

**Figure 1:**
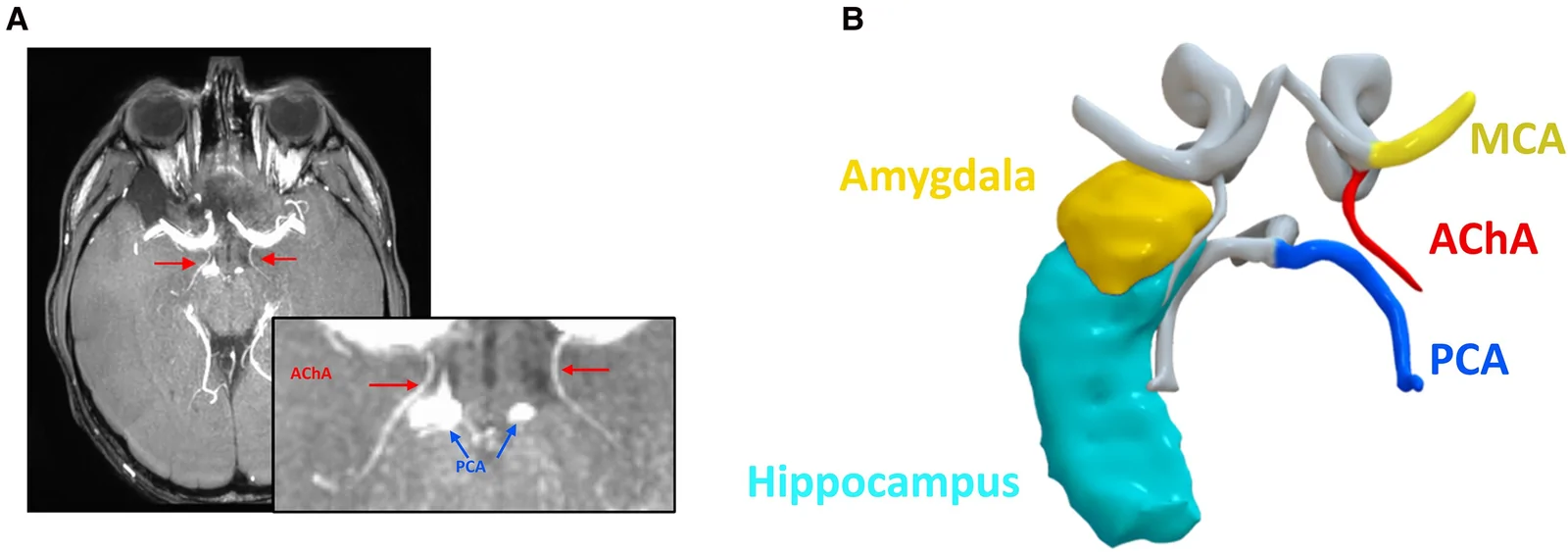
**Medial temporal lobe arteries.** (**A**) Axial TOF-MRI at the level of the AChA and the PCA. (**B**) 3D rendering of the main arteries of the CW segmented with eICAB adjacent to MTL regions. AChA: anterior choroidal artery. MCA: middle cerebral artery. PCA: posterior cerebral artery. TOF-MRA: time-of-flight magnetic resonance angiography. CW: circle of Willis. eICAB: express intracranial arteries breakdown. MTL: medial temporal lobe.

Data from 151 CU participants were analysed (Table 1). We included participants with a clinical dementia rating (CDR) score of 0, and with MRA, tau-PET, amyloid-PET, sociodemographic and health-related information available. Arterial diameter size was extracted from MRA images using eiCAB (express IntraCranial Arteries Breakdown), a fully automated method for accurate artery identification and diameter measurement recently introduced by our group.^30,31^ The arteries of interest in this study were the PCA, the middle cerebral artery (MCA) and AChA as these are commonly reported as being the main arterial supply routes to the MTL^32-34^ (Fig. 1). Therefore, diameter measurements from eICAB were obtained for the M1 segment of the MCA, the P1 segment of the PCA and the cisternal segment of the AChA. As a proxy marker for blood flow,^35^ artery length was estimated by the total number of skeleton voxels for each artery mask. For tau–PET data, images were first registered to their corresponding T1-weighted image and subsequently registered in MNI space. PET data were normalized to standardized uptake value ratios (SUVRs) by dividing each voxel’s intensity by the mean intensity of the entire cerebellum, which served as a reference region. Mean SUVRs of the brain were extracted based on FreeSurfer version 5.3 segmentation. The MTL was defined as the composite region of the amygdala, entorhinal cortex and parahippocampal cortex. The hippocampus region was excluded from the tau analysis because of off-target binding of the tau tracer in the choroid plexus.^36^

**Table 1:** Social and health-related information of CU individuals

	Participants
*N*	151
Sex (% of male)	40%
Mean age (years)	67.6
Mean number of cardiovascular risk factors	1.8
Mean education (years)	16.2
Mean amyloid whole-brain (SUVr)	1.12

The cardiovascular risk factors were recorded as binary variables and included hypertension, diabetes, smoking, obesity and hypercholesterolaemia.

SUVr: relative standardized uptake value.

### Relationship between cognitive decline and artery size

The dataset used for the cognitive decline analysis was also acquired from OASIS.^37^ Information about data acquisitions can be found at: https://sites.wustl.edu/oasisbrains/home/oasis-3/.

MRA images from all participants with a baseline CDR score of 0.5 and at least one longitudinal follow-up were processed using eICAB (*n* = 43). This analysis was only performed on individuals with MCI, as too few participants in the CU group underwent cognitive decline. To assess the rate of cognitive decline, we computed the slope of the CDR-sum of boxes (CDR-SB) score over time (Δ CDR-SB/year). We used CDR-SB instead of CDR because of its greater range of values and utility in accurately tracking changes in cognition.^38,39^ Participants with a CDR-SB pts./year value greater than zero were assumed to be declining (*N* = 23, +1.37 pts./year) while the remaining were assumed to be stable (*N* = 20, −0.45 pts./year). These numbers are consistent with large-scale studies showing that approximately half of MCI patients remain cognitively stable over an ∼2-year period.^40,41^ The two groups did not differ significantly in age, sex, cardiovascular disease and MR image quality (Table 2).

**Table 2:** Social and health-related information of MCI individuals

	Slow decline	Fast decline	*P*-value
*N*	20	23	
Sex (% of male)	50%	43%	0.67
Mean age (years)	72.5	75.7	0.16
Follow-up (years)	1.74	2.26	0.11
Cardiovascular disease (%)	20	26	0.44
Mean education (years)	17.4	14.7	0.002
Amyloid whole-brain (SUVr)	1.34	1.67	0.007

The cardiovascular disease column represents the percentage of participants with at least one cardiovascular disease.

SUVr: relative standardized uptake value.

### Statistical analyses

The mean of the left and right measurements for arterial diameter and tau load was used for statistical analysis. Spearman correlations were calculated to assess the relationship between arterial diameter and tau burden/cognitive decline. Correlation analyses were reported both with and without correcting for confounding factors. Previous studies have shown that age and amyloid burden are associated with artery size and tau burden,^4,9,42^ therefore potentially acting as confounding factors in our correlation analyses. Thus, age and amyloid load were included as covariates in the assessment of tau-artery size relationship. Similarly, because there is evidence that amyloid levels and education are associated with cognitive decline,^43,44^ correcting for these in the cognitive decline analysis was considered appropriate. Since we tested three arterial segments, we employed the Holm–Bonferroni method to correct for multiple comparisons, and the subregion analyses were reported as exploratory. In the cognitive decline study, five participants were excluded from the corrected analysis due to missing amyloid data. Student’s *t*-tests were used to compare arterial diameters between declining and stable MCI participants.

## Results

### Vascular supply to the MTL

**Figure 2:**
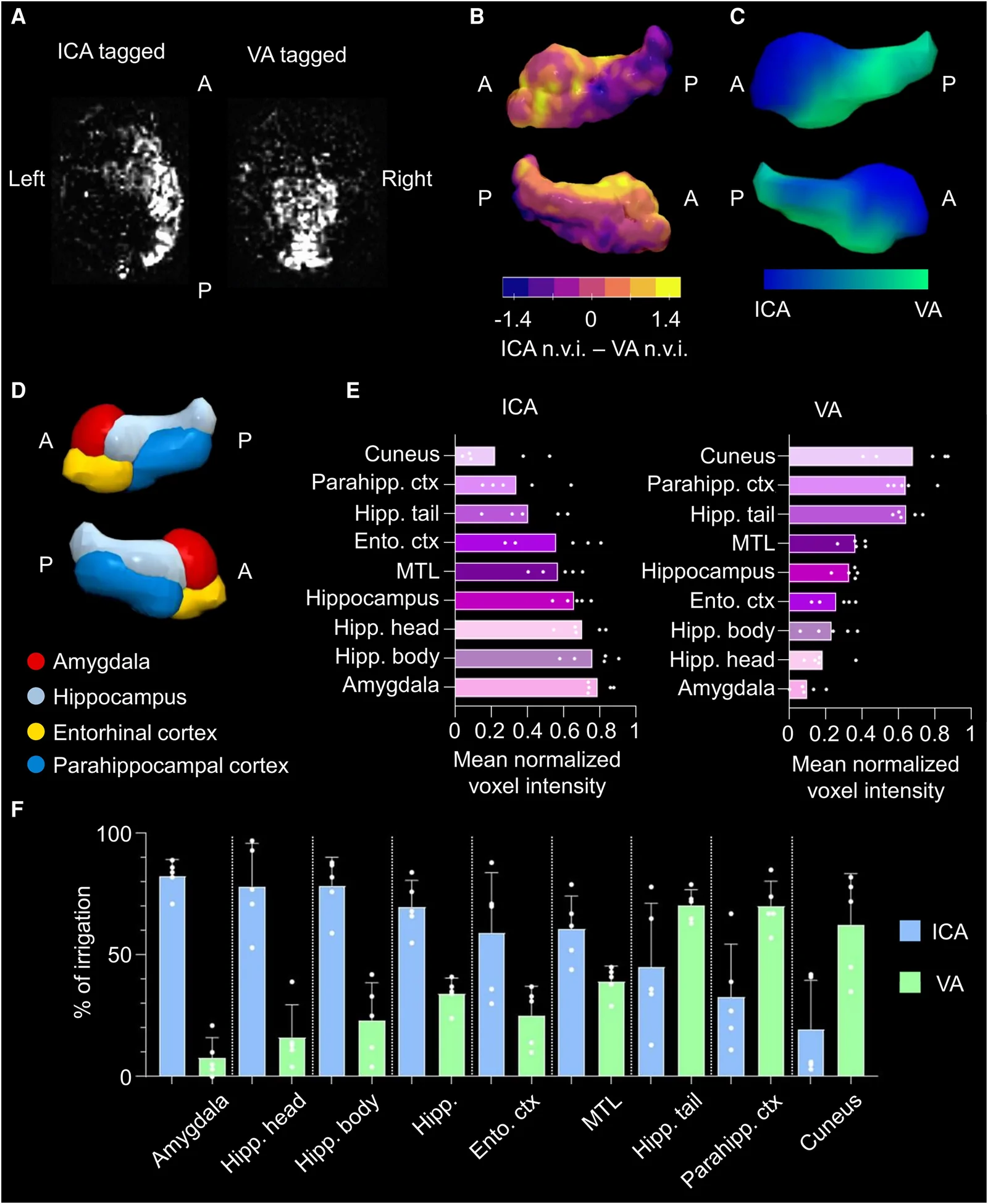
**Supply gradient of the MTL.** (**A**) Example of an ss-ASL image when tagging arterial blood in the right ICA, which is directly beneath the AChA, and the VA. (**B**) **A** representative case of a perfusion map applied to a 3D surface reconstruction of the MTL showing the difference in n.v.i between ICA and VA. (**C**) Similar map as in **B**, but showing voxels meeting a threshold of 0.4 n.v.i for each arteries. (**D**) Corresponding regions of the MTL. (**E**) Mean normalized voxel intensity for each region on ICA-tagged and VA-tagged images (*N* = 5 participants). (**F**) Percentage of the region irrigated by the ICA and the VA based on a 0.4 n.v.i threshold. Each data point represents a participant. n.v.i: normalized voxel intensity. ICA: internal carotid artery. VA: vertebral arteries. A: anterior. *P*: posterior. MTL: medial temporal lobe.

Post-mortem studies^33,45^ indicate that the MTL is supplied by arteries forming in the anterior (MCA, AChA) and posterior circulation (PCA, Fig. 1), yet *in vivo* evidence of their respective contributions has been lacking. To address this, we scanned five young, healthy participants using an ss-ASL sequence (see Methods) to map the areas of the MTL supplied by anterior, posterior or both (mixed) circulations. We chose to scan young participants to ensure that our findings accurately reflected normal perfusion. Older adults might display age-related changes, such as stenosis, that could affect perfusion territories and bias the experiment. An example of an unprocessed ss-ASL image from one participant is shown in Fig. 2A. Group-averaged maps of vascular supply (see Methods) are shown in Fig. 2B-C. MTL regions such as the amygdala and hippocampal head region were almost exclusively perfused by the anterior circulation, whereas parahippocampal cortex and hippocampal tail regions were more primarily supplied by the posterior circulation (Fig. 2D-F). To our knowledge, these findings are the first *in vivo* observations revealing a distinct vascular supply gradient of the MTL, transitioning from anterior circulation supply to the amygdala and hippocampal head, to a posterior circulation supply to the hippocampal tail.

### MTL tau is higher in CUs with small AChAs

**Figure 3:**
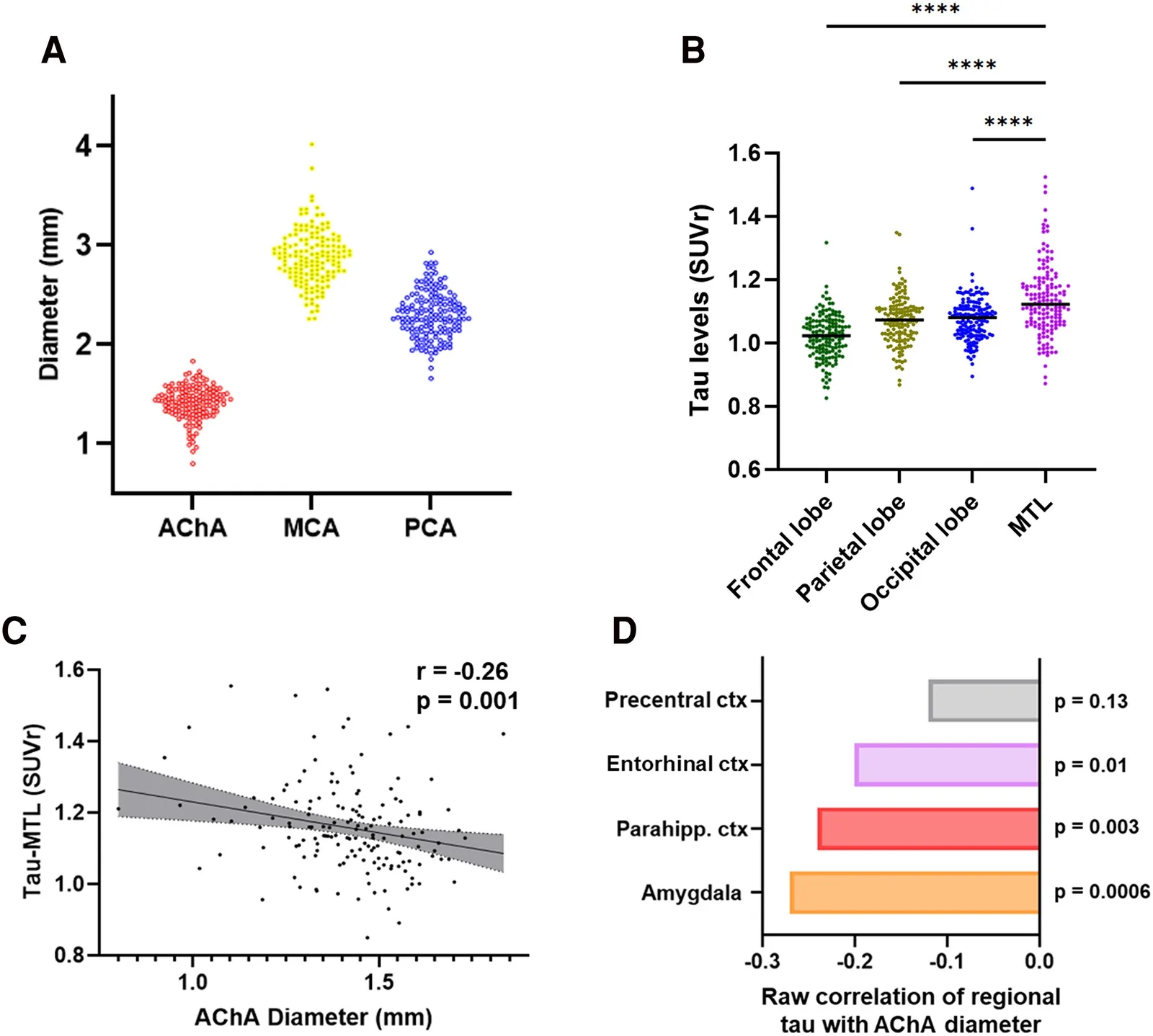
**AChA diameter is associated with MTL tau.** (**A**) Lumen diameter of the CW arteries across a CU population (*N* = 151 participants). The error bar represents the standard deviation. (**B**) Distribution of tau burden for all subjects in major regions of the brain. One-way ANOVA with Tukey’s honestly significant difference test was used to assess differences between regions (F = 122.5, *P* < 0.0001). (**C**) Scatter plots of the association between AChA diameter and tau burden in the MTL. The 95% confidence intervals are represented in grey areas. (**D**) Raw correlation between AChA diameter and regional tau load. In sub-panels C and D, Spearman’s rank correlation was used to assess these relationships. In sub-panel **A**, **B** and **C**, each datapoint represents a participant and values are average of both hemispheres. ctx: cortex. SUVr: relative standardized uptake value. **** : *P* < 0.0001. AChA: anterior choroidal artery. MCA: middle cerebral artery. PCA: posterior cerebral artery.

We next examined whether the calibre of the vessels feeding the MTL is associated with tau levels. To address this, tau–PET and MRA images were analysed in 151 CU individuals (Fig. 3A). On average, tau levels were significantly higher in the MTL compared to the frontal (*P* < 0.0001), parietal (*P* < 0.0001) and occipital cortices (*P* < 0.0001), consistent with the known selective vulnerability of the MTL to tau pathology in early stages (Fig. 3B). MTL tau burden correlated negatively with AChA diameter (r = −0.26, *P* = 0.001, Fig. 3C), but not with MCA or PCA diameter (*P* > 0.8). The association between AChA diameter and MTL tau remained significant after correcting for age, neocortical Aβ levels (r = −0.19, *P* = 0.02) and multiple comparison. Log-transforming the AChA diameter did not alter the association with tau levels. Within the MTL, AChA diameter showed the strongest correlation with tau levels in the amygdala and weakest with tau in the entorhinal cortex (Fig. 3D). We did not find any association between the length of any of our arteries of interest and tau levels in the MTL (all *P* > 0.3), suggesting a flow-independent relationship between AChA diameter and tau levels.

### Cognitive decline is accelerated in patients with small AChAs

**Figure 4:**
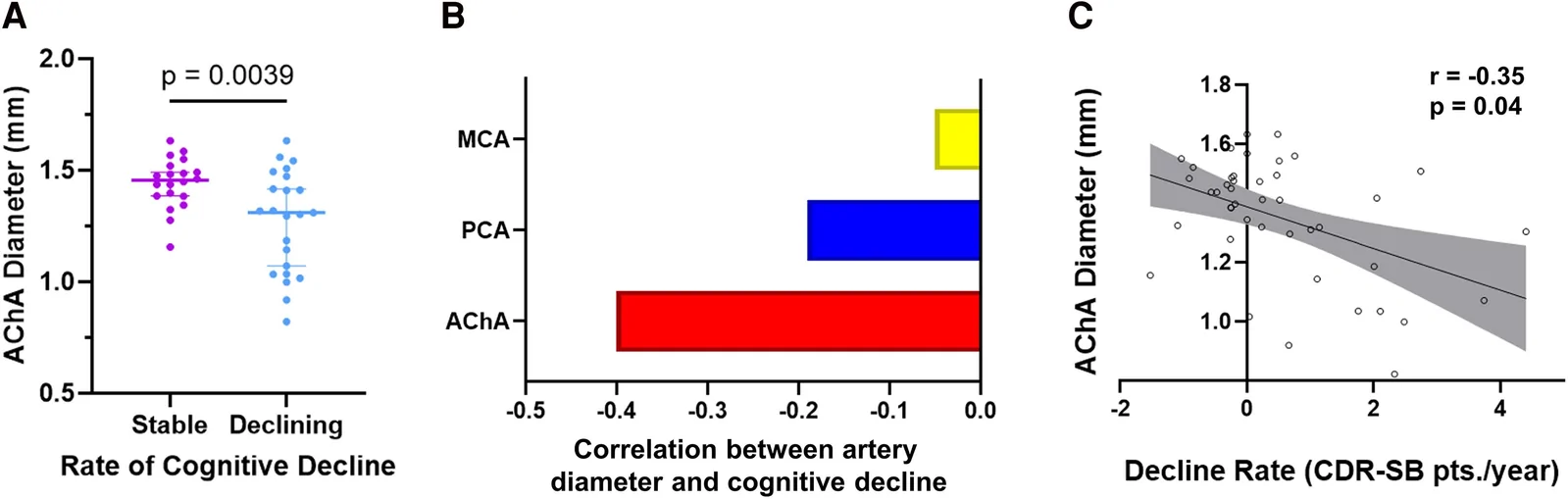
**AChA diameter is associated with the rate of cognitive decline.** (**A**) Average AChA diameters are smaller in declining MCI subjects (*N* = 23 participants) versus stable MCI (*N* = 20 participants). Student’s *t*-test was used to compare both groups (t = 3.059, *P* = 0.0039). (**B**) The correlation between baseline artery diameter and decline rate was significant only for the AChA, not for other CW arteries. (**C**) Same data as in (**A**), but viewed as a scatter plot with correlation adjusted for education and amyloid. Spearman’s rank correlation was used to assess these relationships. In sub-panel **A** and **C**, each datapoint represents a participant. AChA: anterior choroidal artery. MCA: middle cerebral artery. PCA: posterior cerebral artery. CDR-SB pts: clinical dementia rating—sum of boxes points.

Previous studies have shown that higher MTL tau levels in individuals with MCI is associated with accelerated cognitive decline.^46,47^ Given the observed relationship between AChA calibre and MTL tau, we tested whether smaller AChA diameters were also linked to accelerated cognitive decline. To do so, we analysed MRA images in 43 MCI individuals with at least two post-imaging cognitive assessments, allowing us to calculate the rate of cognitive decline, as defined by the yearly change in CDR-SB scores (CDR-SB pts/year) (see Methods). Participants with a CDR-SB pts./year value greater than zero were assumed to be in ‘fast’ cognitive decline (*N* = 23, +1.37 pts./year) while the remaining were assumed to be in ‘slow’ cognitive decline (*N* = 20, −0.45 pts./year). We found that participants with fast cognitive decline had significantly smaller AChA diameters at baseline compared to the stable group (*P* = 0.0039, Fig. 4A). No significant relationships were found between PCA (r = −0.18, *P* = 0.23) or MCA diameters (r = −0.05, *P* = 0.77, Fig. 4B). The correlation between baseline AChA diameter and rate of cognitive decline remained significant after adjusting for education and neocortical Aβ levels (without correcting for confounders: r = −0.40 *P* = 0.012; with correction of confounders: r = −0.35, *P* = 0.04, Fig. 4C). As in the previous analysis, no significant correlation was found between artery length and cognitive decline (all *P* > 0.09).

## Discussion

A remarkable feature of tau deposition is its predictable anatomical progression through the brain. Tau deposits typically first appear in the MTL, then spread in an orderly sequence to limbic, association and finally sensory cortices.^14,48,49^ While several studies have investigated the mechanisms facilitating the spread of tau from the MTL to other brain regions,^50-52^ the reason the MTL is particularly vulnerable to the initial insult remains unclear. Selective vulnerability in neurodegenerative diseases describes the phenomenon whereby specific brain regions or cell types succumb to degeneration earlier and more severely than others. Two theories have been proposed to explain this phenomenon: the neuronal theory, which emphasizes intrinsic neuronal properties, and the vascular theory, which focuses on blood supply and neurovascular interactions.^53^ The vascular theory originated in early 20th century studies of stroke and ischaemia, which highlighted how regions with inherently poor blood supply are the first to succumb to hypoxia and oxidative stress.^26,54^ In contrast, the neuronal theory gained prominence later, driven by advances in molecular biology. It emphasizes intrinsic factors like metabolic stress,^55^ neuroplasticity burden^56^ and gene expression^57^ that render certain neurons particularly vulnerable. Since then, the neuronal theory has become the dominant explanation for selective vulnerability in AD.^15,58^ In this context, the present study brings renewed attention to a vascular-based model of selective vulnerability. We observed that individuals with narrower AChAs exhibited higher MTL tau levels and accelerated cognitive decline. A reduced arterial calibre may reflect a limited perfusion reserve, potentially rendering the MTL tissue more susceptible to metabolic stress and impaired protein clearance, thereby creating an environment that promotes tau formation,^59^ neurodegeneration^24,25^ and ultimately, cognitive decline.

This interpretation is supported by multiple experimental studies showing that ischaemia induces marked increases in pathological tau protein,^60-62^ which is presumably a pathological response to hypoxia-related mechanisms.^20-23^ According to our ss-ASL data and previous post-mortem studies,^33^ the anterior portion of the MTL, which is the initial site of tau accumulation,^63^ is primarily supplied by the AChA. Consistently, correlations between AChA diameter and tau burden were strongest in the amygdala, a region heavily relying on the AChA for blood supply. Overall, these results indicate that inefficient vascular supply may represent an amyloid-independent regulator of the topographical selectivity of early tau protein accumulation.^11,64^

Nonetheless, the precise cause of a narrow AChA is unclear. *Post hoc* analysis revealed a lack of correlation between artery diameter and the number of cardiovascular risk factors, the latter of which are strongly linked to atherosclerotic lesions. In addition, the link between AChA diameter and amyloid burden was very weak. Collectively, these observations imply that AChA calibre may be an inherent interindividual vascular trait marking reduced resilience, rather than a direct consequence of atherosclerotic or amyloid angiopathy-related changes. These variations in AChA calibre may represent a developmentally determined vascular set point, established during early arterial competition, that influences lifelong regional resilience to neurodegenerative processes.

A key strength of this study is that it highlights MRA and vascular morphology analysis^30,65,66^ as important tools for better understanding neurodegenerative disorders. The results from this study, combined with recent advances in non-contrast-enhanced MRA image analysis, underscore the importance of including blood vessel structure in neuroimaging studies. However, certain limitations should be acknowledged. Although our results suggest that vascular factors may underlie selective vulnerability, the directionality of the association between AChA diameter and tau burden remains uncertain. It is possible that tau accumulation leads to loss of function in neurons and reduced firing rates, resulting in lower metabolic demand and, consequently, reduced blood flow through neurovascular coupling. If that is the case, AChA diameter changes would be secondary to tau deposition. Longitudinal studies are therefore needed to assess the temporal sequence between arterial narrowing and tau deposition. Additionally, the lack of available Alzheimer’s disease biomarkers other than PET imaging (i.e. fluid biomarkers) in this dataset limits our ability to further investigate the relationship between blood vessels and tau levels. Future studies using ss-ASL in elderly individuals would also be required to confirm the hypothesis that reduced AChA calibre causes perfusion deficits in the anterior MTL. Finally, the relatively small size of our ss-ASL cohort and MCI cohort reduces statistical power, especially when controlling for multiple confounding variables. Future studies with larger cohorts will be necessary to confirm these findings and further investigate the vascular contribution to early tau pathology.

In a well-characterized population, we found that individuals with narrower AChA had increased tau levels and experienced faster cognitive decline. These results raise the possibility that the selective vulnerability of the MTL in aging and Alzheimer’s disease emerges, at least in part, from Aβ-independent vascular dysfunction.

## Data Availability

eICAB, the artery segmentation and measurement algorithm, is available at the following repository: https://gitlab.com/FelixDumais/vessel_segmentation_snaillab. The OASIS dataset is available upon request. More information at: https://sites.wustl.edu/oasisbrains/request-access/.
